# Does self‐compassion help to deal with dietary lapses among overweight and obese adults who pursue weight‐loss goals?

**DOI:** 10.1111/bjhp.12499

**Published:** 2020-12-24

**Authors:** Cecilie Thøgersen‐Ntoumani, Louisa A. Dodos, Andreas Stenling, Nikos Ntoumanis

**Affiliations:** ^1^ Physical Activity & Well‐Being Research Group School of Psychology Curtin University Perth Western Australia Australia; ^2^ Department of Psychology Umeå University Sweden; ^3^ Department of Sport Science and Physical Education University of Agder Kristiansand Norway

**Keywords:** obesity, dieting, diary study, ecological momentary assessment, temptations, multilevel modelling

## Abstract

**Objectives:**

Self‐compassion can facilitate self‐improvement motivation. We examined the effects of self‐compassion in response to dietary lapses on outcomes relevant to weight‐loss strivings using a longitudinal design. The indirect effects of self‐compassion via guilt and shame were also explored.

**Design:**

An Ecological Momentary Assessment methodology was employed with a sample of adults who were overweight or obese attempting to lose weight via dietary restriction (*N* = 56; *M*_age_ = 34.88; *SD* = 13.93; M*_BMI_* = 32.50; *SD* = 6.88) and who responded to brief surveys sent to their mobile phones twice daily for two weeks.

**Methods:**

Dietary temptations and lapses were assessed at each diary entry, and self‐compassion in response to dietary lapses, intention to continue dieting, weight‐loss‐related self‐efficacy, negative reactions to the lapse, and self‐conscious emotions were surveyed on occasions when participants reported having experienced a dietary lapse. The participants were also weighed in a laboratory prior to the EMA phase and via self‐report straight after the EMA phase. Weight was measured again in the laboratory 12 weeks after the EMA period.

**Results:**

Bayesian multilevel path analyses showed that self‐compassion did not predict weight loss. However, at the within‐person level, self‐compassion was positively related to intentions and self‐efficacy to continue dieting, and negatively related to negative affective reactions to the lapses. Guilt mediated the associations of self‐compassion with intention, self‐efficacy, and negative reactions.

**Conclusion:**

Self‐compassion may be a powerful internal resource to cultivate when dieters experience inevitable setbacks during weight‐loss strivings which could facilitate weight‐loss perseverance.

**Statement of Contribution**

***What is already known on this subject?***

- Dietary lapses often curtail weight‐loss goal attainment.
- Dietary lapses often result in negative emotional reactions.
- Self‐compassion can attenuate negative emotional experiences and promote positive feelings.

***What does this study add?***

- Self‐compassion facilitates weight‐loss goal perseverance at the within‐person level, but did not predict weight loss.
- Reductions in guilt explain these associations.
- Self‐compassion could be a powerful resource to cultivate to help people deal with dietary lapses.

## Background

More than two in three adults are currently classified as overweight or obese (Ng et al., 2014). This is problematic because overweight and obesity are causally implicated in cardiovascular disease, type 2 diabetes, and some cancers (The GBD, 2015 Obesity Collaborators, 2017). In addition to physical health risks, overweight and obesity bear a significant threat to psychological health, including low self‐esteem (Annis, Cash, & Hrabosky, 2004; Carr & Friedman, 2005), depression (Xiang & An, 2015), and anxiety (Hatzenbuehler, Keyes, & Hasin, 2009).

Given the high proportion of overweight and obesity in the general population, unsurprisingly, engagement in personal weight control attempts is highly prevalent (Thompson & Stice, 2001). A meta‐analytic study indicated that 42 per cent of adults in the general population worldwide had engaged in weight‐loss attempts at some point in their lives, with the highest rates reported among those classified as overweight or obese (Santos, Sniehotta, Marques, Carraça, & Teixeira, 2017). These statistics indicate that weight control is a significant issue for the general population, and particularly so for individuals with overweight and obesity.

One of the most commonly adopted behavioural weight‐management strategies is consuming a calorie restricted diet, which when adhered to, can facilitate weight loss (Jeffery et al., 2000). However, consuming a low calorie diet over an extended period of time is challenging (Mann et al., 2007) often resulting in dietary lapses that may curtail weight‐loss goal attainment (Goldstein et al., 2018). Dietary lapses are a product of a complex combination of many different factors including, but not limited to, a) increased hunger (Goldstein et al., 2018; McKee, Ntoumanis, & Taylor, 2014), b) perceived deprivation (Forman et al., 2017; Goldstein et al., 2018), c) self‐control fatigue (i.e., depletion; McKee et al., 2014) and cognitive load (Ward & Mann, 2000), d) mood states (Carels, Douglass, Cacciapaglia, & O’Brien, 2004; Forman et al., 2017; Goldstein et al., 2018), and g) social factors, such as the presence of other people (McKee et al., 2014). Dietary lapses have been negatively correlated with worse weight‐loss outcomes, even when participants do not abandon their weight‐loss efforts altogether (e.g., Forman et al., 2017). Thus, lapses are a key driver of weight‐loss failure. Coping with lapses is critical for preventing further lapses and promoting psychological well‐being when individuals are attempting to lose weight, and help to prevent drop out from weight‐loss efforts (Forman et al., 2017; Goldstein et al., 2018). It is, thus, imperative to enhance understanding of malleable psychological factors that allow individuals to cope with these lapses, and thus persevere with their weight‐loss efforts. Indeed, a conceptual review (Elfhag & Rossner, 2005) and results of observational research (Dohm, Beattie, Aibel, & Striegel‐Moore, 2001) suggests that responses to dietary lapses are a key predictor of weight‐loss maintenance. This research suggests that maintainers, compared to regainers, tend to use direct coping strategies (such as treating relapse as a small mistake, increasing exercise) (Dohm et al., 2001), have better coping strategies, greater ability to handle life stress, higher levels of self‐efficacy, control of over‐eating, autonomy, and psychological strength than regainers (Elfhag & Rossner, 2005). Regainers are more likely than maintainers to eat in response to negative feelings, and react more passively to problems (Elfhag & Rossner, 2005). The main objective of our study is to examine the role of malleable factors.

### Self‐compassion following personal failures

One such malleable factor is self‐compassion. Self‐compassion is a self‐attitude which comprises three interrelated facets (Neff, 2003). These are (a) *self‐kindness*, providing the self with patience, warmth, and understanding in response to perceived personal failure, as opposed to responding with harsh condemnation and *self‐judgement*; (b) *mindfulness*, noticing and observing aversive and difficult to tolerate thoughts and emotions as they arise to create distance and balanced awareness, without becoming excessively immersed in or *over‐identifying* with them, intensifying failures or inadequacies; and (c) *common humanity*, perceiving a moment of suffering as a collective human experience, rather than one that is overwhelmingly *isolating* and disconnecting. Self‐compassion is conceptualized as both an enduring personality trait and a momentary state that can be domain‐ or event‐specific (Leary, Tate, Adams, Batts Allen, & Hancock, 2007).

Terry and Leary (2011) have proposed that it is in situations when people are faced with personal difficulties and failure that self‐compassion comes into play. Indeed, a meta‐analysis of observational studies has shown that self‐compassion can attenuate negative emotional experiences and promote positive feelings (*r *= .15; *p *< .001, with low heterogeneity) (Sirois, Kitner, & Hirsch, 2015). Such affect regulation facilitates effective self‐regulation to persevere with behaviour change consistent with one’s goals. The self‐improvement properties of self‐compassion may be particularly applicable in the domain of weight loss for health improvement. Indeed, the results of a systematic review of six intervention studies lasting between 1 and 5 weeks suggest that self‐compassion may help people lose weight (Rahimi‐Ardabili, Reynolds, Vartanian, McLeod, & Zwar, 2018). Further, a diary study with college women showed that on days when they reported relatively high levels of self‐compassion (compared to other days), they reported significantly more functional eating practices in the form of greater levels of intuitive eating and less dietary restraint (Kelly & Stephen, 2016). However, how individuals responded to self‐regulation failures was not assessed. In instances where people fail to self‐regulate their behaviour to align with a set goal, such as eating a ‘forbidden’ food while on a diet, they often experience negative emotional reactions, including guilt, shame, and self‐criticism (Heatherton, 1993; Polivy, Herman, & Deo, 2010). Such emotional states tax an individual’s self‐regulation capacity, often resulting in the individual giving up on their goals all together (Polivy et al., 2010; Terry & Leary, 2011). When self‐compassionate, individuals may come to accept their momentary dieting failure without experiencing highly self‐critical or defensive reactions. Self‐compassion may also help them to acknowledge a need to reengage in weight‐loss efforts in order to achieve their goals. Although self‐compassion was not assessed, we are aware of one relevant Ecological Momentary Assessment study conducted with adults who were overweight or obese (*N* = 91) (Schumacher et al., 2018). The study examined how self‐attitudes (i.e., forgiving oneself, self‐criticism, positive self‐regard) and self‐efficacy following dietary lapses predicted lapse frequency and risk of lapsing again later on in the same day. The results showed that typical negative self‐regard was significantly associated with greater lapse frequency, and lower momentary self‐criticism negatively and significantly predicted lapses later the same day. No empirical research to date has examined the role of self‐compassion specifically in response to dietary lapse experiences.

### The mediating role of self‐conscious emotions

Understanding factors that may mediate the effects of self‐compassion on weight‐loss‐related outcomes is not only useful conceptually, but it can also serve the practical purpose of identifying mechanisms that can be used as intervention targets. Results of a meta‐analysis examining associations between self‐compassion and health behaviours (including consumptions of healthy diets) found that positive and negative affect significantly mediated these associations in all the studies (*n* = 8) measuring affect (Sirois et al., 2015). We propose that in the context of weight‐loss strivings, it will be valuable to consider self‐conscious emotions (shame and guilt) as mediators in the associations between self‐compassion and outcomes related to weight‐loss perseverance. This is because dietary transgressions are often experienced as failures and are inherently ego‐threatening (Terry & Leary, 2011). In turn, such experiences have been found to significantly predict negative self‐conscious emotions including guilt, shame, and related self‐criticism (Terry & Leary, 2011). These negative self‐focused emotions serve to threaten continued dieting, as the individual may employ defensive reactions leading to impaired decision‐making, or reactive abandonment of weight‐loss goals to provide immediate, but costly, relief from the self‐attack (Adams & Leary, 2007; Muraven, Collins, Morsheimer, Shiffman, & Paty, 2005). Results of an observational study have shown that self‐compassion is significantly and negatively associated with feelings of guilt associated with eating in university students (Wasylkiw, MacKinnon, & MacLellan, 2012). However, the interrelationships between self‐compassion, guilt, and shame have not been explored at the within‐person level nor in the context of dietary lapses. Thus, the within‐person interrelationships between self‐compassion, self‐conscious emotions, and dietary perseverance were examined for the first time in the present study.

While previous studies provide important preliminary evidence to underscore the importance of self‐compassion for positive health behaviours, affect, and self‐regulation, they are limited by the cross‐sectional methodologies used. Naturalistic methodologies, such as Ecological Momentary Assessment (EMA) comprising repeated assessments of constructs of interest over time, are particularly useful to assess the dynamic within‐ and between‐person associations between these constructs following dietary lapses. EMA permits the collection of repeated, immediate data reports of the occurrence of events and feelings in real time, thus reducing retrospective recall bias and increasing ecological validity (Stone & Shiffman, 2002). A small number of studies have employed EMA methods in the context of weight‐loss strivings (Carels et al., 2004; Forman et al., 2017; McKee et al., 2014; Schumacher et al., 2018). These studies have examined daily experiences over 7‐ to 14‐day periods. However, this body of research has focused on examining the factors that influence the occurrence of dietary temptations and lapse episodes, not reactions to dietary lapses. This is an important omission as lapses are often inevitable when striving to lose weight over an extended period of time. Further, intrapersonal coping responses to lapses, such as self‐compassion, that are likely to influence weight‐loss perseverance have not been explored in these studies.

Weight‐loss goal perseverance can be conceptualized in a range of ways. In the current study, in addition to weight loss, we considered intention and self‐efficacy as particularly important indicators of weight goal perseverance. Intention is one of the most widely studied proximal predictors of behaviour (Ajzen & Kuglanski, 2019), with results of meta‐analyses suggesting that intentions predict dietary behaviour (McDermott et al., 2015; McEachan, Conner, Taylor, & Lawton, 2011). Individuals with high levels of self‐efficacy believe they can successfully cope with dietary challenges, and as a result are more like to persevere with their weight‐loss goals (Latner, McLeod, O’Brien, & Johnston, 2013). This argument is supported by empirical evidence showing that self‐efficacy is significantly and positively associated with weight‐loss success (Elfhag & Rössner, 2005; Kitsintas, 2000).

### Aims and hypotheses

We examined if self‐compassion was related to constructs associated with weight‐loss goal perseverance at the between‐ and within‐person level, among adults who were overweight or obese with weight‐loss goals. Our main hypotheses refer to the within‐person level, as we were primarily interested in the daily associations between self‐compassion, self‐conscious emotions, and weight‐loss‐related outcomes. We hypothesized that self‐compassion in response to a dietary lapse would positively predict weight loss (measured in terms of weight‐loss goal achievement at the conclusion of a 14‐day diary period and at 12 weeks; H1). Further, at the within‐person level, state self‐compassion in response to a dietary lapse would be positively related to intentions to continue dieting, and weight‐loss‐related self‐efficacy (H2). In addition, at the within‐person level, self‐compassion would be related to fewer negative reactions to the dietary lapse (H3). Additionally, the hypothesized associations between self‐compassion and the outcomes (intentions, self‐efficacy and negative reactions a dietary lapse) would be mediated by self‐conscious emotions (shame and guilt; H4).

## Method

### Participants and procedure

Participants were a community sample of adults with overweight or obesity who had weight‐loss goals, and who were about to start, or had commenced, their weight‐loss plan within the last month. All participants reported using dietary restriction only, exercising only, or a combination of the two to achieve their weight‐loss goals. Participants were recruited via an undergraduate course credit participation pool and via a staff online weekly newsletter at a University in Western Australia. Further, flyers were posted in local gyms and cafes, and on Facebook. Exclusion criteria were as follows: individuals using pharmacological and surgical weight‐loss methods, pregnant women, individuals with a serious medical or psychological disorder, body mass index (BMI) lower than 25, and people who reported having a current or previous eating disorder diagnosis. Following ethics approval (HRE2017‐0064; 24/02/17) from a local university, adults were recruited by the second author, and participants were asked by her to provide written electronic informed consent prior to completing the initial questionnaire. Study participants (*n* = 56) ranged in age from 17 to 70 years (*M*_age_ = 34.31, *SD* = 14.14). The majority were single (38.60%), full‐time students (41.40%) but many were also employed full‐time (32.90%). Participants described themselves as predominantly Caucasian (78.60%) and to a lesser extent Asian (14.30%) or African (4.30%).

Participants were contacted via email or mobile phone. After providing written informed consent, they completed a battery of online screening items, socio‐demographic questions, and trait measures described below. On a different day, participants came into a research laboratory at the University, to provide height and weight measurements, taken by the research team. Three separate measurements were taken of each participant’s weight and height, using a Tanita weighing scale and a SECA stadiometer, respectively. BMI was calculated from a mean of the three measurements: kg/ height squared (m^2^). Subsequently, the researcher described the procedure for the diary phase. Additionally, verbal and written instructions regarding how to identify dietary temptations and lapses were provided to participants. A temptation was defined as ‘a sudden urge to overeat or consume a forbidden food or drink, in which one felt they had come close to the brink of breaking their diet’ (Carels et al., 2001). A dietary lapse was defined as ‘an incident where you felt that you broke your diet’ (Carels et al., 2001). Participants were instructed to complete two electronic diary entries per day for 2 weeks, which were sent directly to their mobile phones via a text message containing a web‐link to a diary uploaded on Qualtrics. Texts were sent at a randomly selected time within each of the two time blocks, 10 a.m.‐2 p.m. and 4‐8 p.m. The brief diary measures assessed the frequency of dietary temptations and lapses, strengths and nature of temptations, self‐compassion in response to lapses, shame, guilt, intentions to continue dieting, self‐efficacy, and negative reactions (since the last diary entry if completed in the afternoon, or since getting up if completed in the morning). At the conclusion of the two‐week diary period, participants were asked to take a weight measurement the morning of day 14 using their personal weighing scale.

The final phase involved participants returning for a 3‐month follow‐up laboratory session to obtain a final measurement of their weight. During this session, participants were also asked if they believed any factor (e.g., medication changes, other life stresses) impacted their ability to lose weight.

### Measures

#### Control variables

At the between‐person level, we controlled for a range of factors previously shown to be associated with weight‐loss success, including weight cycling and the tendency to engage in dichotomous thinking (Madigan, Pavey, Daley, Jolly, & Brown, 2018; Palascha, van Kleef, & van Trijp, 2015). Further, trait self‐compassion was added as a control variable to assess the contribution of lapse‐specific self‐compassion, above and beyond the generalized tendency to be self‐compassionate.

##### Weight cycling

A single item (‘*How many times in your life have you purposefully lost 4.5 kgs (10 pounds) or more and regained it*?’) assessed participants’ history of weight cycling. Participants’ responses were scored on an 11‐point scale ranging from 0 to 10, where higher scores reflect greater levels of previous instances of weight cycling (the Global Burden of Metabolic Risk Factors of Chronic Diseases Collaborating Group (Body Mass Index et al., 2012).

##### Dichotomous thinking

The tendency to engage in all‐or‐none thinking was measured using the 11‐item Dichotomous Thinking in Eating Disorders Scale (Byrne, Allen, Dove, Watt, & Nathan, 2008). The items were measured on a 4‐point scale from 1 (*Not at all true of me*) to 4 (*Very true of me*). An example item is ‘I think of myself as either doing very well or very badly’.

##### Trait self‐compassion

Trait self‐compassion was assessed using the *Self‐Compassion Scale‐Short‐Form* (SCS‐S) (Raes, Pommier, Neff, & Van Gucht, 2011), which captures the three facets of self‐compassion described in the introduction. The SCS‐SF contains 12 items (e.g., ‘When I fail at something important to me I become consumed by feelings of inadequacy’). Responses were made using a 5‐point scale (1 = *almost never*; 5 = *almost always*) reversing items where relevant, with higher scores indicating higher levels of trait self‐compassion.

#### Between‐person measures

##### Weight loss

Participants’ weight at baseline and week 12 was taken by the research team; for logistic reasons, participants self‐reported their weight at the end of the EMA phase (start of week 3). The 2‐week weight loss was determined by subtracting weight at 2 weeks from weight at baseline. The same formula was used to calculate weight loss at week 12 (i.e., by subtracting week 12 weight from the baseline weight).

#### Within‐person measures (Diary)

##### Dietary temptations and lapses

Items assessed the occurrence and number of dietary temptations and lapses, the nature of and the reasons for the dietary lapses. Participants were required to indicate whether they had experienced any temptations by selecting *yes* or *no*, and the number of temptations experienced (‘*If yes, how many?’)*. Further, participants were asked to consider the strength of their most recent temptation, which was assessed using a scale ranging from 1 (*not very*) to 7 (*extremely*). Subsequently, participants were asked whether they had lapsed by selecting *yes* or *no* (‘*Have you acted on any of these temptations?’)*, and the number of lapses. The participants were also asked about the nature of their most dietary lapse (‘*Consider the most recent instance when you acted on your dietary temptation…what food did you give into temptation to?*’). If participants indicated no lapse, they received no further questions at that specific time.

##### Self‐compassion in response to lapses

To reduce participant burden, a 3‐item version of the SCS‐SF was employed to assess participants’ level of state self‐compassion in response to their most recent dietary lapse (Raes et al., 2011). Three items were selected based on their content validity: the over‐identification item ‘*I have been obsessing and fixating over breaking my diet*’, the self‐kindness item ‘*I have been understanding about breaking my diet’*, and the self‐judgement item ‘*I have been judgmental about breaking my diet*’.

##### Intention to continue dieting

Two items (‘*I am determined to continue to diet in the next two weeks’, ‘I intend to diet in the next two weeks’)* were embedded in the diary to assess participants’ intentions to engage in dieting behaviour in the next two weeks, following their most recent dietary lapse. These items were scored on a 7‐point scale ranging from 1 (*strongly disagree*) to 7 (*strongly agree*). The items were adapted from the two‐item measure used in previous research (Thøgersen‐Ntoumani & Ntoumanis, 2006). Intentions are commonly assessed using two items only.

##### Self‐efficacy

State self‐efficacy for continuing to eat healthful foods was assessed using an adapted version of the *Brief Self‐Efficacy Scales for Use in Weight‐Loss Trials* measure (Wilson et al., 2016). A sample item is ‘How confident are you that you can stick to eating healthful foods?’. Participants’ self‐efficacy ratings were made on an 11‐point scale, from 0% to 100%.

##### Negative reactions to lapse occurrence

To tap rigid, black, and white cognitive style towards dietary lapses at the state level, two items (‘*I feel like giving up my diet plan*’, ‘*I feel I have failed my diet plan*’) were included in the diary. These items were chosen as they were relevant to assess at the state level, whereas the remaining items were not. The items were adapted from the *Dichotomous Thinking in Eating Disorders Scale* (DTEDS) (Byrne et al., 2008). The items were scored using a scale ranging from 1 (*strongly disagree*) to 6 (*strongly agree*).

##### Self‐conscious emotions

State shame and guilt were measured using the 15‐item *State Shame and Guilt Scale (SSGS)* (Marschall, Sanftner, & Tangney, 1994). Participants were instructed to consider their feelings in relation to their most recent lapse, which were scored on a 5‐point scale ranging from 1 (*not feeling this way at all*) to 5 (*feeling this way very strongly*). Items were adapted to reflect their most recent lapses, for example, ‘I feel bad about breaking my diet’. The SSGS has been successfully used in previous diary research (Conroy, Ram, Pincus, & Rebar, 2015).

### Statistical analysis

We used Mplus version 8.2 to estimate Bayesian multilevel path analyses at the between‐ and within‐person levels (Muthén & Muthén, 1998‐2017). State self‐compassion, shame, guilt, intention, self‐efficacy, and negative reactions were within‐person level variables. Age, weight cycling, trait self‐compassion, dichotomous thinking, sex, and the between‐person component of state self‐compassion were between‐person level variables. The between‐person level variables were included as covariates in all models. Latent mean centring was used for the within‐person level variables, whereas the between‐level predictors were grand‐mean centred (Asparouhov & Muthén, 2019). A 95% credibility interval was calculated for each parameter estimate in the models. The credibility interval indicates the probability (e.g., 95%) that the parameter of interest lies between the lower and upper bound of the interval. Furthermore, if the 95% CI did not include zero, the parameter estimate was considered to be a credible parameter estimate (i.e., we could reject the null hypothesis of no effect; Zyphur & Oswald, 2015). For more details about this analysis, please see Data S1.

Bayesian multilevel mediation analysis (Yuan & MacKinnon, 2009) was performed and evaluated using 95% highest posterior density intervals, which accommodate the asymmetry in the distribution of the mediated effect (Miočević, MacKinnon, & Levy, 2017). If an interval did not include zero, we concluded that the indirect effect was credible and statistically significant. In all analyses, we relied on the default non‐informative prior specification in Mplus. Reliability estimates are presented as omega coefficients (ω) for scales with three or more items, whereas for the 2‐item scales we calculated coefficient alpha (Geldhof, Preacher, & Zyphur, 2014).

## Results

### Preliminary analyses

#### Sample

**Figure 1:**
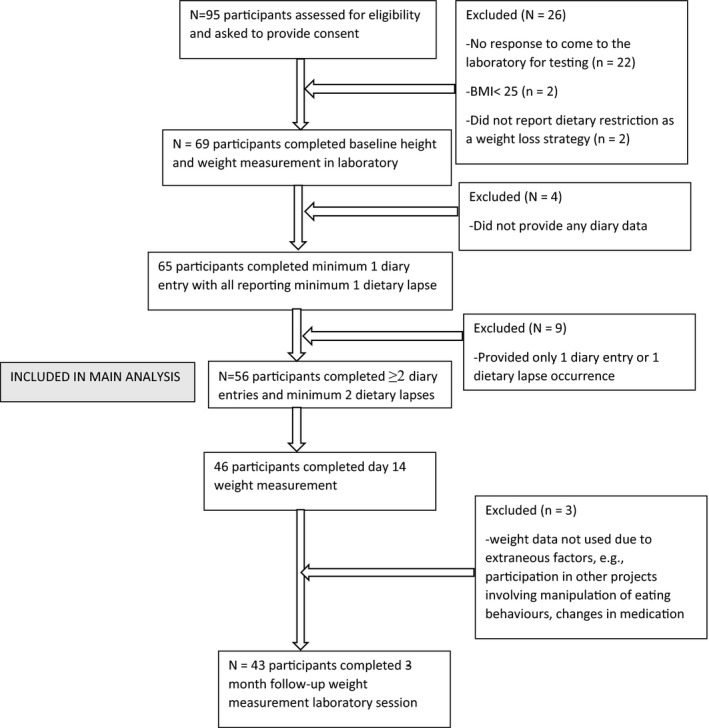
Participant Flow (STROBE).

We only included participants who had experienced at least one dietary lapse during the two weeks of the diary study, and reported that they were using dietary restriction to lose weight. This resulted in a sample size of 56 participants (4 males, 52 females; *M*_age_ = 34.88, *SD* = 13.93). Figure 1 presents an overview of the participant flow. Our sample size at the between level is around the lower limit for estimating fixed effects in multilevel SEM. For example, Hox and McNeish (2020) suggested that around 50 subjects are required for fixed effects in this type of model. Note that this is not a universal recommendation because required sample sizes are influenced by many factors. Our main interest, however, was in the daily associations (i.e., the within‐person level coefficients) for which we had 254 observations. Limited recommendations in the literature have been provided related to sample sizes in 1‐1‐1 multilevel mediation models with Bayesian estimation and fixed effects. However, based on the available literature (e.g., Hox & McNeish, 2020; see also Zigler & Ye, 2019, for simulations on 1‐1‐1 multilevel mediation models with random effects), we deemed our within‐person level sample size to be sufficient for estimating the (fixed) indirect effects. The average weight loss was 1.87 kg (*SD* = 1.64, observed range + 0.30 to −6.20) after 2 weeks and 1.05 kg (*SD* = 3.20, observed range + 6.20 to −17.30) after 12 weeks. Eight participants (14.3%) lost ≥ 5% of their baseline weight at the 12‐week follow‐up.

#### Compliance

Across the 28 electronic diaries, the average compliance rate was 30.8% (*SD* = 16.0%). The compliance rate includes answering yes or no to the question ‘Have you acted on any of these temptations (an instance when you broke your diet)?’. About half of these responses were yes responses (16.2%), which resulted in a total of 254 data points (participants x occasions) at the within‐person level. The average BMI before commencing the diary study was 32.50 (*SD* = 6.88).

#### Temptations and lapses

Participants recorded 477 dietary temptations across the two‐week diary study and they reported acting on (i.e., lapsing) 366 of those temptations. The average strength of the dietary temptations was 5.67 (*SD* = 1.43), which indicates that the participants experienced quite strong dietary temptations. The most frequently reported type of dietary temptation was convenience food (26.8%; e.g., fast food, take away food), the second most common type was biscuits/cake/dessert (26.4%), and the third most common type was chocolate in various forms (12%). Other less commonly reported temptations included alcohol, bread, candy, cheese, chips, ice cream, and soft drinks.

#### Correlations, intra‐class correlation coefficients, reliability coefficients, and descriptives

Within‐ and between‐person level correlations, intra‐class correlation (ICC) coefficients, reliability coefficients, means, and standard deviations are presented in Table 1. The ICCs ranged from 0.41 to 0.70 indicating that there was meaningful variability at the between‐ and within‐person level.

**Table 1:** Between (above the diagonal)‐ and Within‐person (below the diagonal) level correlations, ICCs, and descriptive statistics

	1	2	3	4	5	6	7	8	9	10	11	12	13
1. WL 2 weeks		.65^**^	−.16	−.24	.03	−.24	.30*	−.10	.08	−.06	.05	.06	.20
2. WL 12 weeks			.01	−.14	−.02	−.18	.27*	−.18	.02	−.17	−.22	−.19	.22
3. Age				.23	.05	.12	.11	.02	−.05	−.13	.18	.02	−.13
4. Weight cycling					−.02	.13	−.02	.11	.07	−.07	−.02	.09	.11
5. Self‐compassion (Trait)						−.29*	.07	.00	.16	−.24	−.37^**^	−.21	.29*
6. Dichotomous thinking							.22	−.10	−.36^**^	.27*	.27*	.32*	−.36^**^
7. Sex								−.02	−.22	.03	−.09	.06	.03
8. Intentions									.65^**^	−.62^**^	−.29*	−.28*	.37^**^
9. Self‐efficacy								.49^**^		−.63^**^	−.27*	−.25	.38^**^
10. Negative reactions								−.56^**^	−.57^**^		.66^**^	.63^**^	−.63^**^
11. Shame								−.33*	−.37^**^	.46^**^		.77^**^	−.71^**^
12. Guilt								−.36^**^	−.42^**^	.53^**^	.69^**^		−.77^**^
13. Self‐compassion (State)								.23	.34^**^	−.49^**^	−.52^**^	−.68^**^	
ICC								.50^**^	.70^**^	.48^**^	.62^**^	.52^**^	.41^**^
Reliability	NA	NA	NA	NA	.88^b^	.75^b^	NA	.59/.74^c^	NA	.52/.62^c^	.72/.94^d^	.76/.91^d^	.69/.82^d^
*M* ^a^	1.87	1.05	34.89	2.54	3.00	2.62	NA	6.11	6.69	2.77	1.81	2.48	4.54
*SD* ^a^	1.64	3.20	13.81	2.60	.65	.70	NA	.85	1.90	1.11	.65	.64	.90

Note

WL = weight loss, NA = not applicable, ICC = intra‐class correlation.

^a^ Based on estimates after missing data handling.

^b^ Omega coefficient (ω).

^c^ Within‐level/between‐level alpha coefficients (α).

^d^ Within‐level/between‐level omega coefficients (ω).

* *p* < .05; **p < .01.

### Bayesian multilevel path analysis

#### Does state self‐compassion predict weight loss?

State self‐compassion was not a credible predictor of weight loss after 2 weeks or 12 weeks (Table 2). None of the between‐level covariates had credible effects on weight loss.

**Table 2:** State self‐compassion as predictor of weight loss. Unstandardized parameter estimates are presented

	Weight loss	
	2 weeks	12 weeks
	Estimate [95% CI]	Estimate [95% CI]
Within‐person level		
Self‐compassion (State)		
Residual variance	1.16 [0.94, 1.40]^a^	1.16 [0.94, 1.40]^a^
*R* ^2^	NA	NA
Between‐person level		
Intercept	−1.29 [−6.07, 3.49]	−3.48 [−12.68, 5.14]
Self‐compassion (State)	0.17 [−0.58, 0.89]	0.29 [−1.59, 2.22]
Age	−0.01 [−0.06, 0.03]	0.03 [−0.07, 0.13]
Weight cycling	−0.02 [−0.29, 0.26]	−0.15 [−0.74, 0.45]
Self‐compassion (Trait)	−0.19 [−1.08, 0.72]	−0.96 [−2.90, 0.92]
Dichotomous thinking	−0.51 [−1.38, 0.37]	−0.77 [−2.45, 0.92]
Sex	2.59 [−1.09, 6.24]	3.49 [−0.85, 7.87]
Residual variance	2.49 [1.41, 4.18]^a^	10.58 [5.62, 18.15]^a^
*R* ^2^	29.7%	25.4%

Note

^a^ 95% CI did not include zero. Self‐compassion (State) is the between‐person component of daily state self‐compassion scores.

Abbreviation: NA = not applicable.

#### Is state self‐compassion related to intentions to continue weight‐loss goal‐directed behaviour, self‐efficacy, and negative reactions after a dietary lapse?

At the within‐person level, state self‐compassion was related to intentions, self‐efficacy, and negative reactions (Table 3). These results indicate that at measurement points when self‐compassion was higher than usual (i.e., compared to the participants’ own mean trajectory), the participants reported greater intentions to continue dieting, self‐efficacy, and less negative reactions following dietary lapses. At the between‐person level, state self‐compassion was negatively associated with negative reactions, suggesting that participants who on average reported higher state self‐compassion also reported less negative reactions. There were no credible relations between any of the other between‐level covariates with intentions, self‐efficacy, or negative reactions.

**Table 3:** Relations between state self‐compassion and intentions to continue dieting, self‐efficacy, and negative reactions following dietary lapse. Unstandardized parameter estimates are presented

	Intentions	Self‐efficacy	Negative reactions
	Estimate [95% CI]	Estimate [95% CI]	Estimate [95% CI]
Within‐person level			
Self‐compassion (State)	0.19 [0.08, 0.30]^a^	0.39 [0.23, 0.55]^a^	−0.52 [−0.66, −0.39]^a^
Residual variance	0.71 [0.58, 0.86]^a^	1.43 [1.16, 1.74]^a^	1.06 [0.86, 1.28]^a^
*R* ^2^	5.5%	11.1%	23.2%
Between‐person level			
Intercept	4.73 [2.73, 6.74]^a^	5.09 [1.05, 8.94]^a^	5.86 [3.68, 8.02]^a^
Self‐compassion (State)	0.35 [−0.05, 0.76]	0.67 [−0.12, 1.47]	−0.77 [−1.21, −0.33]^a^
Age	0.00 [−0.02, 0.03]	0.00 [−0.04, 0.04]	−0.02 [−0.04, 0.01]
Weight cycling	0.02 [−0.09, 0.13]	0.04 [−0.18, 0.26]	0.01 [−0.11, 0.13]
Self‐compassion (Trait)	−0.12 [−0.59, 0.33]	0.08 [−0.83, 0.99]	−0.09 [−0.58, 0.41]
Dichotomous thinking	−0.04 [−0.49, 0.42]	−0.61 [−1.50, 0.27]	0.10 [−0.41, 0.59]
Sex	−0.17 [−1.33, 0.99]	−1.49 [−3.76, 0.79]	0.43 [−0.83, 1.70]
Residual variance	0.87 [0.47, 1.41]^a^	3.74 [2.13, 5.82]^a^	0.97 [0.50, 1.58]^a^
*R* ^2^	20.0%	24.5%	41.5%

Note

^a^ 95% CI did not include zero. Self‐compassion (State) is the between‐person component of daily state self‐compassion scores.

#### Do shame and guilt mediate the relation between state self‐compassion and intentions to continue weight‐loss goal‐directed behaviour, self‐efficacy, and negative reactions following a dietary lapse?

In Table 4, we present the results when adding state shame and guilt to the model as mediators between state self‐compassion and intentions, self‐efficacy, and negative reactions following dietary lapses. At the within‐person level, state self‐compassion was related to shame and guilt, whereas only guilt was related to intentions, self‐efficacy, and negative reactions. The results indicate that at measurement points when participants reported higher self‐compassion than usual they also reported less shame and guilt than usual. In addition, at measurement points when they reported more guilt than usual they also reported lower intentions, self‐efficacy, and more negative reactions. Guilt mediated the relation between self‐compassion and intentions, self‐efficacy, and negative reactions following dietary lapses, whereas no credible indirect relations were observed through shame (Table 5).

**Table 4:** Relations between state self‐compassion, shame, and guilt and intentions to continue dieting, self‐efficacy, and negative reactions following dietary lapse. Unstandardized parameter estimates are presented

	Shame	Guilt	Intentions	Self‐efficacy	Negative reactions
	Estimate [95% CI]	Estimate [95% CI]	Estimate [95% CI]	Estimate [95% CI]	Estimate [95% CI]
Within‐person level					
Self‐compassion (State)	−0.24 [−0.30, −0.19]^a^	−0.39 [−0.45, −0.33]^a^	−0.03 [−0.17, 0.12]	0.11 [−0.10, 0.32]	−0.23 [−0.40, −0.06]^a^
Shame			−0.26 [−0.57, 0.05]	−0.37 [−0.80, 0.08]	0.35 [−0.01, 0.72]
Guilt			−0.39 [−0.68, −0.10]^a^	−0.50 [−0.92, −0.08]^a^	0.54 [0.19, 0.90]^a^
Residual variance	0.19 [0.16, 0.24]^a^	0.21 [0.17, 0.26]^a^	0.65 [0.53, 0.79]^a^	1.33 [1.07, 1.61]^a^	0.94 [0.76, 1.14]^a^
*R* ^2^	26.5%	45.5%	15.1%	19.2%	32.7%
Between‐person level					
Intercept	4.20 [3.27, 5.17]^a^	5.06 [4.20, 5.95]^a^	5.24 [0.41, 10.33]^a^	3.38 [−6.68, 13.22]	2.58 [−2.46, 7.40]
Self‐compassion (State)	−0.53 [−0.75, −0.33]^a^	−0.57 [−0.76, 0.38]^a^	0.28 [−0.40, 0.99]	0.86 [−0.51, 2.27]	−0.39 [−1.08, 0.29]
Shame			−0.27 [−1.14, 0.62]	−0.35 [−2.06, 1.42]	0.80 [−0.07, 1.68]
Guilt			0.10 [−0.91, 1.11]	0.60 [−1.36, 2.65]	0.03 [−0.96, 1.03]
Age			0.01 [−0.02, 0.03]	0.01 [−0.04, 0.05]	−0.02 [−0.04, 0.00]
Weight cycling			0.02 [−0.01, 0.14]	0.03 [−0.21, 0.26]	0.01 [−0.12, 0.13]
Self‐compassion (Trait)			−0.17 [−0.66, 0.32]	0.00 [−0.96, 0.97]	0.05 [−0.43, 0.55]
Dichotomous thinking			−0.03 [−0.49, 0.44]	−0.60 [−1.51, 0.31]	0.08 [−0.39, 0.55]
Sex			−0.18 [−1.37, 1.04]	−1.56 [−3.87, 0.85]	0.49 [−0.72, 1.71]
Residual variance	0.24 [0.13, 0.39]^a^	0.19 [0.10, 0.31]^a^	0.87 [0.47, 1.44]^a^	3.80 [2.17, 6.06]^a^	0.83 [0.42, 1.39]^a^
*R* ^2^	49.1%	58.0%	27.0%	29.3%	54.4%

Note

^a^ 95% CI did not include zero. Self‐compassion (State) is the between‐person component of daily state self‐compassion scores.

**Table 5:** Indirect relations between self‐compassion and intentions to continue dieting, self‐efficacy, and negative reactions through shame and guilt. Unstandardized parameter estimates are presented

	Estimate (*ab*)	95% CI
Within‐person level		
Self‐compassion (State) → Shame → Intentions	.06	[−0.01, 0.14]
Self‐compassion (State) → Shame → Self‐efficacy	.09	[−0.02, 0.20]
Self‐compassion (State) → Shame → Negative Reactions	−.08	[−0.18, 0.01]
Self‐compassion (State) → Guilt → Intentions	.15	[0.04, 0.27]^a^
Self‐compassion (State) → Guilt → Self‐efficacy	.19	[0.03, 0.37]^a^
Self‐compassion (State) → Guilt → Negative Reactions	−.21	[−0.36, −0.07]^a^
Between‐person level		
Self‐compassion (State) → Shame → Intentions	.14	[−0.35, 0.63]
Self‐compassion (State) → Shame → Self‐efficacy	.18	[−0.80, 1.16]
Self‐compassion (State) → Shame → Negative Reactions	−.41	[−0.94, 0.07]
Self‐compassion (State) → Guilt → Intentions	−.06	[−0.66, 0.52]
Self‐compassion (State) → Guilt → Self‐efficacy	−.34	[−1.58, 0.79]
Self‐compassion (State) → Guilt → Negative Reactions	−.01	[−0.61, 0.56]

Note

^a^ 95% CI did not include zero. Self‐compassion (State) is the between‐person component of daily state self‐compassion scores. *ab* = indirect effect.

At the between‐person level, state self‐compassion was related to guilt and shame, suggesting that people who on average reported higher self‐compassion reported lower shame and guilt. No other direct or indirect relation involving self‐compassion was credible at the between‐person level (see Table 4 and Table 5). None of the other between‐level covariates had a credible relation with intentions, self‐efficacy, or negative reactions.

## Discussion

This is the first study to examine the role of self‐compassion for outcomes associated with weight‐loss goal perseverance. In doing so, we used an EMA methodology that allows daily associations to be examined at the within‐person level, controlling for between‐person differences.

The findings did not show support for the first hypothesis, in that participants’ aggregate of state level of self‐compassion in response to a dietary lapse did not significantly predict weight loss during the 2‐week EMA study period nor at the 12‐week follow‐up. This finding diverges from results of a recent systematic review of intervention studies which showed that self‐compassion promotes weight loss, although importantly only three of six studies included in the review examined objective weight loss (Rahimi‐Ardabili et al., 2018). The lack of weight change after week 2 in our study could have been attributed to the self‐reporting of weight at week 3, and thus measurement bias. It is also likely explained by the short time frame. Although the 12‐week follow‐up period was selected as the minimum realistic time period for the achievement of moderate weight loss, this time period may also have been too short. Indeed, clinically significant weight loss, usually operationalized as 5% or higher reductions in initial body weight (Williamson, Bray, & Ryan, 2015), was not achieved by the majority of participants in this study. Results of a large study conducted in the UK revealed that the median amount of weight lost following participation in a three‐month commercial weight‐loss intervention programme was 3.1% from initial weight, with only a third of dieters losing ≥ 5% weight (Ahern et al., 2017). Thus, it may be beneficial in future research to examine these associations using a longer weight‐loss period, particularly in EMA studies with no intervention component, as was the case with ours.

Further, we found that self‐compassion can alleviate the negative reactions associated with self‐regulation failure. The results showed that when participants reported higher than average levels of state self‐compassion following a dietary lapse, they reported greater intentions to continue dieting, higher levels of self‐efficacy to eat healthily, and less negative reactions to the lapse, supporting H2 and H3. Thus, in moments when individuals felt highly self‐compassionate, they appeared better able to frame their momentary lapse occurrence in a balanced way, so as to not endorse negative, and self‐defeating thinking in relation to their diet. Further, participants were able to simultaneously report a greater belief in their ability to reengage with dieting goal despite their dietary setback. Indeed, previous research demonstrated the importance of framing dietary lapses as transient states, so as to positively predict future self‐efficacy to resist, rather than give up and give in to inevitable temptations (McKee et al., 2014). Further, a qualitative investigation of successful and unsuccessful weight maintainers showed that one of the variables that discriminated the two groups in favour of the successful group was good coping skills with dietary failures (McKee, Ntoumanis, & Smith, 2013). Our results are consistent with conceptualizations of self‐compassion as a positive internal coping resource in the face of momentary experiences of failure (Breines & Chen, 2012). Self‐compassion can help dieters reengage with dietary regulation after failure by reductions in negative feelings and the promotion of positive feelings (see meta‐analysis by Sirois et al., 2015), which, in turn, can counteract ego depletion and facilitate self‐regulation (Tice, Baumeister, Shmueli, & Muraven, 2007). Being kind (ie., compassionate) to oneself in moments of ‘weakness’ may help individuals revert to intuitive eating because self‐compassion helps individuals being more aware of body signals (Kelly & Stephen, 2016). Lastly, self‐compassion can reduce ego threats, and hence alleviate feeling of defeatism and hopelessness associated with the lapse, thus protecting psychological resources (Johnson & O’Brien, 2013).

The results revealed partial support for the mediation hypotheses (H4). Specifically, reductions in feelings of guilt partly explained the associations between self‐compassion in response to dietary lapses and the outcomes related to goal perseverance at the within‐person level. This is an important finding, as guilt is associated with a lack of control and less weight‐loss success (Kuijer & Boyce, 2014). Feelings of guilt leave dieters feeling defeated and hopeless about their capacity to lose weight and continue dieting, increasing the risk of total diet abandonment (Carels et al., 2001, 2004). Our finding aligns with past research showing that self‐compassion can reduce feelings of guilt associated with eating (Braun, Park, & Gorin, 2016; Breines, Toole, Tu, & Chen, 2014). We extend past research to show that reductions in guilt associated with applying self‐compassion may explain positive outcomes in adults with overweight and obesity who actively pursue weight loss. H4 was not fully supported, because although self‐compassion was negatively related to shame following dietary lapse, shame was not related to intentions, self‐efficacy, and negative reactions. Nonetheless, our findings make a unique conceptual contribution to existing literature on the role of self‐compassion to dietary perseverance by identifying guilt reduction as a possible mechanism via which self‐compassion is related to goal perseverance in a weight‐loss context.

### Strengths and limitations

Strengths of our study include the use of an EMA methodology to examine two previously untested research questions, namely the relation between self‐compassion and weight‐loss‐related responses to dietary lapses, and whether self‐conscious emotions mediate such processes. Although prior research has shown that inducing self‐compassion may help individuals lose weight (Rahimi‐Ardabili et al., 2018) and eat in more functionally adaptive ways (Kelly & Stephen, 2016), our study is the first of its kind to demonstrate the role of self‐compassion in helping adults deal with dietary setbacks. Our findings are important because they have shown that being self‐compassionate can help individuals persevere in their weight‐loss efforts, rather than give up all together. Indeed, persevering is critical for weight‐loss success. Self‐compassion may also reduce the risk of excessive weight cycling which is associated with weight gain and depressive symptoms (Madigan et al., 2018). Thus, the results suggest that future programmes designed to help overweight and obese individuals lose weight should incorporate self‐compassion training. In a similar vein, nutritionists and health care providers interacting with individuals with weight‐loss goals should be encouraged promote a self‐compassionate attitude in their patients/clients to help them deal with negative emotions experienced during their weight‐loss journeys. Additional strengths of the study include the assessment of BMI at two measurement occasions, surveying adults with overweight or obesity starting a weight‐loss journey, and the rigorous statistical analysis employed (Bayesian path analysis).

Some limitations should be considered in interpreting the study’s results. Firstly, due to the use of the signal contingent methodology, the dietary lapses participants experienced may have taken place up to several hours before the signal, thus potentially resulting in some memory bias. It might be useful in future research to employ a mix of signal and event contingent sampling to partly address this limitation. Second, the mean compliance rate (30.8%) was quite low. One possible explanation for the low compliance is that the participants were students or employed, hence they might have been occupied with other things and unable to respond to some of the prompts when they received them. In fact, a recent study on non‐compliance with EMA studies found that 70% of participants who missed to respond to a prompt stated that the reason was not being able to do so (Eisele et al., in press). Third, it is possible that factors not measured in the present study could impact how participants responded to dietary lapses. These factors include physical activity, sleep, dietary adherence, and perceived sustainability of the individual’s eating plan. Finally, the severity of the dietary lapse (i.e., the amount of food consumed) or whether it was deliberate (‘I will eat this cake because I haven’t brought lunch’) or impulsive (‘I will eat this cake although I know I shouldn’t’) was not assessed. It may be interesting in future research to assess possible differences in coping and emotional responses depending on the severity and degree of impulsivity of lapses, and whether the effects of self‐compassion on the assessed outcomes differs depending on lapse severity and impulsivity. In addition, it would be interesting in future research to experimentally induce self‐compassion (e.g., via a mobile app) (Rodgers et al., 2018) following a dietary lapse to examine its effects on the outcomes examined in the present study. Self‐compassion can be induced via Neff’s mindful self‐compassion programme (Neff, 2009), which has been shown to produce improvements in self‐compassion (medium effect sizes) (Palmeira, Pinto‐Gouveia, & Cunha, 2017; Rahimi‐Ardabili, Vartanian, Zwar, Sharpe, & Reynolds, 2020; Rodgers et al., 2018). Self‐compassion could be an added component in self‐regulatory training programmes for dietary temptations (McKee & Ntoumanis, 2014). Finally, our results suggest that there may be merit in targeting guilt in future interventions. For example, inducing mindfulness when encountering dietary setbacks, so that individuals learn to accept a lapse and allowing it to exist may go some way in reducing feelings of guilt in response to the setback (Brewer et al., 2018).

### Conclusions

The present study is the first to employ an EMA methodology to examine the within‐person interrelationships between self‐compassion, self‐conscious emotions (guilt and shame), and dietary perseverance. We did not find that self‐compassion experienced on a daily basis predicted objective weight loss in adults pursuing weight loss up to 12 weeks later. Importantly, we did find that self‐compassion was positively related to important weight‐loss goal perseverance outcomes via lower than usual feelings of guilt. Thus, our results suggest that when dieters experience inevitable setbacks during weight‐loss strivings, self‐compassion may be a powerful internal resource to cultivate to promote positive intentions to continue dieting, foster high levels of self‐efficacy, and reduce negative reactions to lapses.

## Conflicts of interest

The authors declare that they have no conflicts of interest.

## Funding

No funding was received for this study.

## Author contributions

Cecilie Thogersen‐Ntoumani (Conceptualization; Data curation; Methodology; Project administration; Resources; Supervision; Validation; Writing – original draft; Writing – review & editing) Louisa A. Dodos (Conceptualization; Data curation; Investigation; Methodology; Writing – original draft; Writing – review & editing) Andreas Stenling (Data curation; Formal analysis; Methodology; Software; Visualization; Writing – review & editing) Nikos Ntoumanis (Conceptualization; Methodology; Project administration; Supervision; Writing – review & editing).

## Ethical approval

All procedures in the study were in accordance with the ethical standards of the Institution and with the 1964 Helsinki declaration and its later amendments or comparable ethical standards.

## Supporting information

Data S1 Details about the data analysis.

## Data Availability

The data that support the findings of this study are available from the corresponding author (first author), upon reasonable request.
