# Supplementary material for: Does self‐compassion help to deal with dietary lapses among overweight and obese adults who pursue weight‐loss goals?
Source: Br J Health Psychol. 2020 Dec 24;26(3):767–88. doi: 10.1111/bjhp.12499 (PMC8451927; doi:10.1111/bjhp.12499)
Supplement: Supplementary file 1 — Data S1 Details about the data analysis. [file BJHP-26-767-s001.docx]

**Supplementary File 1.**

Latent mean centering provides a clear separation of the between and within effects and provides more accurate estimates and standard errors, compared to the traditional observed variable mean centering (Tihomir Asparouhov & Muthén, 2019). The Bayesian multilevel models were estimated using four Markov chain Monte Carlo chains and 50,000 iterations. The first 25,000 iterations were discarded as burn-in and the remaining 25,000 iterations were used to estimate the posterior distribution of the parameters. Chain convergence was assessed using the potential scale reduction factor (Brooks & Gelman, 1998), where a low (e.g., < 1.05) and stable PSFR was considered as evidence of chain convergence. Model fit was evaluated using the posterior predictive *p* (PPP) value and its accompanying 95% confidence interval. A PPP values around 0.50 with a 95% CI centering on zero was considered as a well-fitting model, whereas a low PPP value and a 95% CI with a positive lower limit was considered a poor fitting model (T. Asparouhov & Muthén, 2010). Parameter estimates were evaluated using the 95% credibility intervals. If an interval did not include zero, the parameter estimate was considered as credible and statistically significant (Zyphur & Oswald, 2015).

**References**

Asparouhov, T., & Muthén, B. O. (2010). *Bayesian analysis using Mplus: Technical implementation. Mplus Technical Report*. Muthén & Muthén.

Asparouhov, Tihomir, & Muthén, B. (2019). Latent Variable Centering of Predictors and Mediators in Multilevel and Time-Series Models. *Structural Equation Modeling: A Multidisciplinary Journal*, *26*(1), 119–142. https://doi.org/10.1080/10705511.2018.1511375

Brooks, S. P., & Gelman, A. (1998). General methods for monitoring convergence of iterative simulations. *Journal of Computational and Graphical Statistics*, *7*, 434–455.

Zyphur, M. J., & Oswald, F. L. (2015). Bayesian Estimation and Inference: A User’s Guide. *Journal of Management*, *41*(2), 390–420. https://doi.org/10.1177/0149206313501200
